# Epidemiology, therapy, and outcome of immune-mediated thrombotic thrombocytopenic purpura at population level in Germany

**DOI:** 10.1016/j.rpth.2026.103406

**Published:** 2026-03-04

**Authors:** Anna Y. Scharenberg, Jakob C. Voran, Sarah-Yasmin Thomsen, Anka Pohlmeyer, Victor Walendy, Linus A. Völker, Roland Schmitt, Friedrich A. von Samson-Himmelstjerna, Kevin Schulte, Benedikt Kolbrink

**Affiliations:** 1 Department of Nephrology and Hypertension, University Hospital Schleswig-Holstein, Campus Kiel, Kiel, Germany; 2 Department of Internal Medicine III, Cardiology and Critical Care, University Hospital Schleswig-Holstein, Campus Kiel, Kiel, Germany; 3 DZHK (German Centre for Cardiovascular Research), partner site Hamburg/Kiel/Lübeck, Kiel, Germany; 4 Department of Internal Medicine II, Medical Faculty of the Martin-Luther-University Halle-Wittenberg, Halle, Germany; 5 Department II of Internal Medicine and Center for Molecular Medicine Cologne, Faculty of Medicine and University Hospital Cologne, University of Cologne, Cologne, Germany

**Keywords:** epidemiology, outcome, therapy, thrombotic microangiopathy, thrombotic thrombocytopenic purpura

## Abstract

**Background:**

Immune-mediated thrombotic thrombocytopenic purpura (iTTP) is a rare, life-threatening thrombotic microangiopathy caused by severe *a disintegrin and metalloproteinase with thrombospondin motifs 13* (ADAMTS-13) deficiency due to autoantibodies. Although therapeutic advances have improved outcomes, data on population-level trends in epidemiology, therapy, and outcomes remain limited.

**Objectives:**

We assessed temporal trends in incidence, treatment strategies, and acute in-hospital outcomes of iTTP episodes at the population level in Germany from 2011 to 2021.

**Methods:**

This retrospective population-based cohort study used nationwide inpatient data from the German Federal Statistical Office. iTTP cases were identified by International Classification of Disease, Tenth Revision, coding combined with documentation of plasma exchange procedures. Trends were analyzed using linear regression.

**Results:**

A total of 2771 iTTP episodes were identified. Incidence of iTTP episodes increased from 2.36 to 3.48 per million inhabitants (*P* = .003), peaking in women aged 30 to 34 and men aged 60 to 64 years. While in-hospital mortality remained stable at 12.8%, complications such as multiorgan failure (33.5%) and need for mechanical ventilation (17.9%) increased significantly. Treatment patterns shifted: the median number of plasma exchange sessions decreased (from 12 to 6), while rituximab use increased (from 24% to 40%, *P* < .001). Caplacizumab was used in 34.7% of patients in 2020 to 2021. Male patients were older and had more severe courses. Outcomes were comparable between university and nonuniversity hospitals.

**Conclusion:**

The incidence and severity of iTTP episodes have increased in Germany over the past decade, and treatment strategies have evolved. These findings warrant further research to optimize acute management and understand long-term outcomes.

## Essentials

• Immune thrombotic thrombocytopenic purpura (iTTP) is a rare, life-threatening blood disorder.
• We analyzed all iTTP hospital cases in Germany from 2011 to 2021.
• Numbers of iTTP cases and severe complications increased, but death rates stayed stable over time.
• Specific treatment of iTTP shifted to fewer plasma exchanges and more targeted drugs.

## 1 Introduction

Immune-mediated thrombotic thrombocytopenic purpura (iTTP) is a rare disease in the heterogenous group of thrombotic microangiopathies (TMAs). Its pathophysiology is characterized by a severely reduced activity of *a disintegrin and metalloproteinase with thrombospondin motifs 13* (ADAMTS-13) caused by autoantibodies. This results in the formation of ultralarge von Willebrand factor (VWF) multimers, which are cleaved by ADAMTS-13 in healthy individuals. Uncleaved VWF excessively binds platelets and thus causes microthrombi and subsequent life-threatening TMA in acute iTTP episodes [1].

Until the 1980s, iTTP episodes were associated with massive mortality, which only changed significantly after therapeutic plasma exchange (PEX) in combination with corticosteroids was established as standard therapy [[2], [3], [4]]. A further improvement in therapy, in particular, the reduction of relapses, was achieved through the additional introduction of the anti-CD20 antibody rituximab for the treatment of iTTP [[5], [6], [7]]. In 2019, caplacizumab, an anti-VWF immunoglobulin fragment that prevents the interaction of VWF and platelets, was shown to be effective in the acute treatment of iTTP in a phase 3 clinical trial, whereupon it entered clinical use [[8], [9], [10]].

The rarity of iTTP has 2 implications. First, the evidence base for the various treatment options is poor [11], and second, the disease is typically treated in specialized centers, between which treatment regimens may differ. Currently, there is no comprehensive overview of the epidemiology, treatment behavior, and outcomes of iTTP episodes at the population level. In addition, further epidemiologic questions about iTTP remain unanswered, particularly the proportion of patients treated outside of specialized centers and potential differences in the course of the disease between men and women.

To answer these questions, we analyzed a database of all inpatient hospital cases in Germany. We generated data on the evolution of the epidemiology, treatment approaches and acute outcomes of iTTP episodes in and outside of specialized centers over a course of 11 years between 2011 and 2021.

## 2 Methods

For this nationwide population-based cohort study, we used the Diagnosis-Related Groups (DRGs) statistics of the German Federal Statistical Office (GFSO), in which comprehensive data on all inpatient hospital cases in Germany are available. We accessed data from January 1, 2011, to December 31, 2021, using controlled remote data processing as described earlier [12,13]. The DRG statistics include information on all inpatient cases in Germany, which German hospitals are obliged to provide annually. Available data included diagnoses and procedures coded according to the International Classification of Diseases, Tenth Revision, German Modification (ICD-10-GM) and the German Operation and Procedure Classification System (OPS), as well as further patient- and hospital characteristics at the single case level in all German hospitals.

To detect inpatient cases with acute episodes of iTTP, we queried the database for all inpatient cases that had the ICD-10-GM code for iTTP (M31.1) and at least one documented PEX (OPS code 8-820). Cases that included relevant diagnoses for potential other causes of TMA like different forms of hemolytic uremic syndrome, systemic lupus erythematosus, and TMAs other than iTTP associated with pregnancy were excluded from the analyses. From the remaining cases of iTTP episodes, further information on demographics, clinical course, and complications were analyzed. An overview of all ICD-10-GM and OPS codes used in the analyses is available in Supplementary Tables 1 and 2. Patients treated at university hospitals were identified by hospital institution codes (Supplementary Table S). Overall, age-specific, and sex-specific incidence rates were calculated based on the population data from the GFSO available via the GENESIS platform [14].

### 2.1 Statistical analysis

All queries, calculations, plots, and statistics were computed using R (R version 4.3.2) with the packages *tidyverse*, *readxl*, and *sf*. We reported continuous variables with median and IQR and categorical variables in absolute and relative frequencies. The chi-squared test was used to compare categorical variables between groups. To assess trends over time, we applied linear regression analyses with beta (β) coefficients and 95% CIs. Results were considered statistically significant with a *P* value <.05.

### 2.2 Ethics statement

This study has been approved by the Research Data Centre of the GFSO (65189 Wiesbaden, Gustav-Stresemann-Ring 11, Germany, forschungsdatenzentrum@destatis.de).

## 3 Results

### 3.1 The incidence of iTTP-episodes in Germany increased significantly from 2011 to 2021

**Figure 1:**
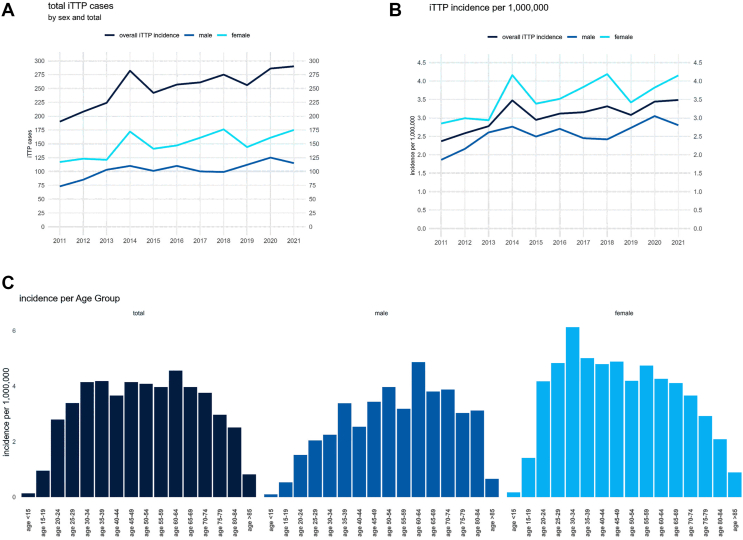
Total, sex-, and age-specific incidence of patients with acute immune-mediated thrombotic thrombocytopenic purpura (iTTP) in German hospitals between 2011 and 2021. (A) Annual raw numbers of acute iTTP episodes treated in German hospitals during the observation period stratified by overall, male, and female patients. (B) Annual overall and sex-specific incidence of acute iTTP episodes per 1,000,000 Germans during the observation period. (C) Age-specific incidence of acute iTTP episodes in Germany stratified by age and sex per 1,000,000 Germans during the observation period.

During the observation period from 2011 to 2021, we identified a total of 2771 acute episodes of iTTP. The incidence of hospital-treated iTTP episodes in Germany increased from a minimum of 2.36 per 1,000,000 inhabitants in the year 2011 to a maximum of 3.48 per 1,000,000 inhabitants in 2021 (Figure 1A, B). This corresponds to an increase of 0.09 per 1,000,000 inhabitants per year (95% CI, 0.04-0.14; *P* = .003). There were substantial differences in the incidence of hospital-treated iTTP episodes between the different German federal states (Supplementary Figure 1). The median age of patients with iTTP was 50 years and did not change significantly over time (Supplementary Figure 2). The most common comorbidity of patients with iTTP was diabetes mellitus at 15.6% followed by cerebrovascular disease (Table 1), and the comorbidities also showed no significant trends over time except for the number of patients suffering from congestive heart failure, which slightly increased (Supplementary Figure 3). No particular seasonal accumulation of iTTP cases (Supplementary Figure 4) was observed. Approximately half of the patients with iTTP were treated in German university hospitals (*n* = 1535, 55.4%), and there were no relevant differences between the hospital types in terms of demographics or disease burden (Table 2).

**Table 1:** Baseline characteristics of patients with immune-mediated thrombotic thrombocytopenic purpura in German hospitals 2011-2021.

Characteristics	Values (*N* = 2771)
Comorbidities	
Congestive heart failure	323 (11.7)
Coronary artery disease	304 (11.0)
Peripheral artery disease	39 (1.4)
Diabetes mellitus	433 (15.6)
Chronic lung disease	77 (2.8)
Cancer	324 (11.7)
Cerebrovascular diseases	405 (14.6)
Dementia	28 (1.0)
Depression	117 (4.2)
In-hospital course	
Myocardial infarction	121 (4.4)
Vascular occlusion	44 (1.6)
Stroke	264 (9.5)
Renal replacement therapy	693 (25.5)
Multiorgan failure	929 (33.5)
Ventilation	495 (17.9)
ICU	1371 (49.5)
Total in-hospital mortality	354 (12.8)
Therapy	
PEX sessions, mean, median (IQR)	9.2, 8 (7)
Rituximab in-hospital, *n* (%)	985 (35.5)

Data are reported as *n* (%) unless otherwise stated.

ICU, intensive care unit; PEX, plasma exchange.

**Table 2:** Patients with immune-mediated thrombotic thrombocytopenic purpura in German hospitals 2011-2021: non-university vs university hospitals.

Characteristics	Hospital type		*P* value
	Nonuniversity	University	
Full cohort	1236 (44.6)	1535 (55.4)	
Female	716 (57.9)	922 (60.1)	.27
Age (y), median (IQR)	51 (37-63)	49 (35-61)	
Comorbidities/complications			
Congestive heart failure	172 (13.9)	151 (9.8)	*.001*
Coronary artery disease	144 (11.6)	160 (10.4)	.33
Peripheral artery disease	20 (1.6)	19 (1.2)	.49
Diabetes mellitus	204 (16.5)	229 (14.9)	.28
Chronic lung disease	34 (2.8)	43 (2.8)	1
Cancer	144 (11.7)	180 (11.7)	1
Cerebrovascular diseases	215 (17.4)	190 (12.4)	*<.001*
Dementia	16 (1.3)	12 (0.8)	.25
Depression	48 (3.9)	69 (4.5)	.48
Therapy			
Rituximab	379 (30.7)	606 (39.5)	*<.001*
PEX sessions, mean, median (IQR)	8.2, 7 (6)	9.9, 8 (8)	
In-hospital course			
Length of stay (d), median (IQR)	20 (10-25)	25 (11-30)	
Myocardial infarction	49 (4.0)	72 (4.7)	.40
Vascular occlusion	15 (1.2)	29 (1.9)	.20
Stroke	146 (11.8)	118 (7.9)	*<.001*
Renal replacement therapy	265 (21.4)	428 (27.9)	*<.001*
Multiorgan failure	426 (34.4)	503 (32.8)	.37
Ventilation	228 (18.4)	267 (17.4)	.50
ICU	647 (52.3)	724 (47.2)	*.008*
Total in-hospital mortality	163 (13.2)	191 (12.4)	.60

Data are reported as *n* (%) or median (IQR) unless otherwise stated.

Itacilized *P* values indicate statistically significant results.

ICU, intensive care unit; PEX, plasma exchange.

### 3.2 Two peaks in incidence: young women and older men

We found a 1.4 times higher incidence in the female population during the observation period (mean male incidence vs mean female incidence 2.55 vs 3.56 per 1,000,000 inhabitants, *P* < .001), although an increase of incidence in both sexes was observed (Figure 1B). The overall sex proportion was 59.1% female and 40.9% male, but the age distribution by sex differed significantly (Figure 1C). The median age of female patients with iTTP was 5 years lower than that of males (48 vs 53 years), and males exhibited a higher prevalence of congestive heart failure, coronary artery disease, peripheral artery disease, diabetes mellitus, chronic lung disease, and cancer compared with females (Table 3). The female incidence peaked in the age group of 30 to 34 years (6.11 per 1,000,000 inhabitants). The highest incidence in males was found in the age group of 60 to 64 years (4.86 per 1,000,000 inhabitants).

**Table 3:** Male vs female patients with immune-mediated thrombotic thrombocytopenic purpura in German hospitals 2011-2021.

Characteristics	Sex		*P* value
	Male	Female	
Full cohort	1133 (40.9)	1638 (59.1)	
Age (y), median (IQR)	53 (40-64)	47.5 (34-61)	
Comorbidities/complications			
Congestive heart failure	158 (13.9)	165 (10.1)	*.002*
Coronary artery disease	152 (13.4)	152 (9.3)	*<.001*
Peripheral artery disease	24 (2.1)	15 (0.9)	*.01*
Diabetes mellitus	211 (18.6)	222 (13.6)	*<.001*
Chronic lung disease	37 (3.3)	40 (2.4)	.24
Cancer	150 (13.2)	174 (10.6)	*.04*
Cerebrovascular diseases	215 (17.4)	190 (12.4)	*<.001*
Dementia	21 (1.9)	7 (0.4)	*<.001*
Depression	41 (3.6)	76 (4.6)	.22
Therapy			
Rituximab	355 (31.3)	630 (38.5)	*<.001*
PEX sessions, mean, median (IQR)	8.5, 6 (7)	9.5, 7 (8)	
In-hospital course			
Length of stay (d), median (IQR)	24 (10-30)	27 (10-27)	
Myocardial infarction	48 (4.2)	73 (4.5)	.85
Vascular occlusion	20 (1.8)	24 (1.5)	.64
Stroke	111 (9.8)	153 (9.3)	.74
Renal replacement therapy	355 (31.3)	338 (20.6)	*<.001*
Multiorgan failure	426 (37.6)	503 (30.7)	*<.001*
Ventilation	226 (19.9)	269 (16.4)	*.02*
ICU	533 (47.0)	838 (51.2)	*.04*
Total in-hospital mortality	157 (13.9)	197 (12.0)	.17

Data are reported as *n* (%) or median (IQR) unless otherwise stated.

Itacilized *P* values indicate statistically significant results.

ICU, intensive care unit; PEX, plasma exchange.

### 3.3 Increasing complication rates in iTTP episodes

**Figure 2:**
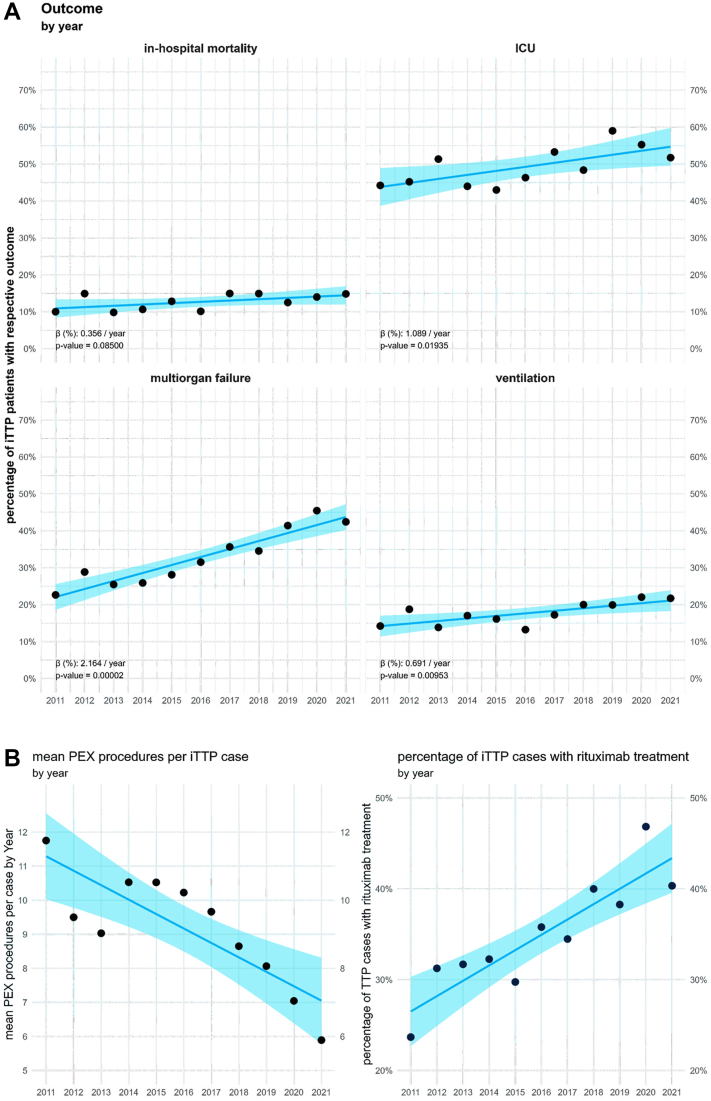
In-hospital outcomes and therapy of patients with acute immune-mediated thrombotic thrombocytopenic purpura (iTTP) in German hospitals between 2011 and 2021. (A) Annual in-hospital mortality and proportions of patients with acute iTTP with intensive care unit (ICU) admissions, multiorgan failure, and in need of mechanical ventilation. Linear regression analyses showed significant trends toward more severe courses in all outcomes except for in-hospital mortality during the observation period. (B) Annual mean numbers of plasma exchange (PEX) procedures performed per acute iTTP episode and proportion of patients with acute iTTP treated with rituximab during their respective hospital stay. Linear regression analyses showed significant trends toward less use of PEX and more use of rituximab during the observation period.

The median treatment duration of patients with iTTP in hospitals was 17 days and did not change significantly over time. The in-hospital mortality rate for all cases was 12.8% and also did not change significantly during the observation period (Figure 2A). Although there were no substantial differences in in-hospital mortality or complication rates between university and non-university hospitals, the median length of stay in university hospitals was 5 days longer than that in other hospitals (Table 2). There were significant differences between men and women in terms of complication rates during the observation period. Males were more likely to require renal replacement therapy, to be in need of mechanical ventilation, and to experience multiorgan failure, although female patients with iTTP had a longer median length of stay. In-hospital mortality was slightly lower in females, although the difference was not statistically significant (Table 3).

Importantly, there was a significant increase in the proportion of patients with iTTP treated in intensive care units (from 44% in 2011 to 51% in 2021; β = 1%/y; *P* = .02), in the proportion of mechanically ventilated patients (from 14% in 2011 to 22% in 2021; β = 0.7%/y; *P* = .02) and in the proportion of patients with multiple organ failure (from 23% in 2011 to 42% in 2021; β = 2%/y; *P* < .001) (Figure 2A).

### 3.4 Decreasing PEX quantity and increasing rituximab application in patients with iTTP

During the observation period, patients with iTTP received a median of 8 PEX sessions during their hospital stay. Rituximab was administered to 36% of patients with iTTP. In university hospitals, patients with iTTP were treated significantly more frequently with rituximab and received more PEX sessions than those in non-university hospitals (Table 2). Furthermore, female patients with iTTP were significantly more often treated with rituximab and received more PEX sessions than men (Table 3). Data on the use of caplacizumab was available for the years 2020 and 2021. In these 2 years, 34.7% (200 of 576) of the patients with iTTP received caplacizumab treatment. The use of caplacizumab was almost twice as high in female patients (40.5%, 136 of 336 patients) than in men (26.7%, 64 of 240 patients).

When analyzing the iTTP treatment over time across all hospitals, we found a significant decrease in mean PEX sessions per iTTP episode (from 12 in 2011 to 6 in 2021; β = −0.42/y; 95% CI, −0.64 to −0.21; *P* = .002). In addition, significantly more patients received rituximab (from 24% in 2011 to 40% in 2021; β = 1.69%/y; 95% CI, 1.04 to 2.33; *P* < .0001) (Figure 2B).

## 4 Discussion

Our analysis of all acute iTTP episodes treated in German hospitals between 2011 and 2021 has 4 main findings: (1) The incidence of treated iTTP episodes steadily increased during the observation period. (2) The number of clinical complications of iTTP episodes increased, while at the same time the treatment regimens changed to less PEX and more rituximab. (3) Male patients with iTTP in Germany are on average older and have more severe courses than females. (4) There are no significant differences in outcomes between university hospitals and other hospitals.

In our study, we observed an incidence of 3.1 hospitalized iTTP episodes per 1,000,000 inhabitants in Germany over a total of 11 years. The annual incidence of iTTP reported in the literature to date on the basis of reliable registry data varies internationally between 2 and 6 per 1,000,000 inhabitants [15,16]. Remarkably, we herein for the first time report a significant increase in the incidence of iTTP episodes at the population level. The reason for this is not clear from our study. We consider an increase in the relapse rates of iTTP to be unlikely, as we were also able to show that more rituximab was administered, which has been proven to lead to a reduction in relapses [5,17,18]. We therefore assume that improved awareness of the condition led to an increase in diagnosis and subsequently in the observable incidence.

We also observed an increase in complication rates and more severe courses, although in-hospital mortality did not change during the observation period. The mortality rate we found for iTTP episodes was 12.8%, which is at the upper end of the range of reported iTTP mortality rates, which to date have varied widely between approximately 1% and over 15% [16]. We assume that we observed a higher mortality rate in our cohort because we conducted a comprehensive survey of all inpatient cases in Germany, which is not subject to selection bias and therefore does not exclude poor outcomes. Surprisingly, the tendency toward a more severe course was not due to a change in the patient population, as the age and comorbidities of the iTTP patients remained stable over time. However, there was a change in the treatment behavior for the iTTP episodes that we observed: the mean number of PEX sessions performed fell from 12 to 6, while the proportion of patients treated acutely with rituximab increased significantly. This could be an expression of a changed understanding of the treatment of iTTP, in which the importance of specific immunosuppressive therapies is increasingly coming to the fore and PEX is being used with increasing caution.

The fact that there was nevertheless a tendency toward more severe courses contradicts earlier reports stating that the early use of rituximab results in better outcomes with fewer PEX sessions required [5,19]. Unfortunately, it is not possible to analyze individual cases on the basis of our data, and there is no information available on further follow-up after discharge from hospital. Nonetheless, this is the first description of changes in iTTP therapy and population-level trajectories over time. Further developments should therefore be monitored, and it will be interesting to see how new treatment options affect the course of iTTP in the future.

Furthermore, we found that male patients with iTTP were older than females and tended to have more severe courses. Women were most frequently affected in the fourth decade and men in the seventh decade of life. It has long been known that iTTP occurs more frequently in women, which is attributed to the female predisposition to autoimmune diseases in general and hormonal changes during pregnancy [1,20,21]. This accumulation at childbearing age is presumably the reason for the lower average age of female patients with iTTP. The reason for the more severe course that we observed in men, however, is most likely their older age, which has been described as an independent risk factor for a more severe course of iTTP [[22], [23], [24]].

Lastly, in this study, we examined the differences between university hospitals and nonuniversity hospitals in the treatment of iTTP. This is particularly interesting as iTTP is generally considered a rare condition that should be treated in specialized centers. Consequently, almost all reports on the course of the disease and treatment come from these centers. Our analysis shows for the first time that a significant proportion of iTTP episodes in Germany are treated outside university hospitals, which are usually considered maximum care providers specializing in rare diseases. Although there were significant differences in the prescribed therapies and the length of stay of patients with iTTP in the different hospital types, the course of the disease did not differ between university hospitals and other clinics. Even if we cannot clarify the causes of these findings at the individual case level, our analysis shows that iTTP is by no means a rare disease that concentrates on maximum care providers, and that awareness of this condition must also exist elsewhere.

### 4.1 Conclusion

We report here for the first time epidemiologic data at the population level on iTTP over a decade and have found that the incidence of diagnosed acute episodes is increasing. We were also able to show that treatment behavior changed significantly during the observation period, while at the same time, the complication rate tended to increase. In addition, a relevant proportion of iTTP episodes are not treated in specialized centers, so awareness of the disease seems to be necessary everywhere. Whether new treatment options will significantly change the treatment and outcomes of iTTP in the future remains to be determined.

### 4.2 Limitations

This was a retrospective analysis of data that were not primarily collected for scientific purposes. Weaknesses of the database are that only data on inpatient hospital stays are available, and there is no follow-up outside the hospital. In addition, it is not possible to validate the results at individual case level, and since we also had no access to laboratory data, we were unable to verify the ADAMTS-13 activity of individual cases. Nevertheless, we analyzed a comprehensive dataset of all hospital cases in Germany, as German hospitals are obliged to report their data to the German DRG statistics annually, therefore excluding selection bias. Furthermore, patient characteristics, such as age and gender distribution, incidence of the disease, and number of PEX sessions performed, are consistent with previously published data on iTTP, which is why we consider the basic analytical approach to be valid [16].
