# Supplementary material for: Epidemiology, therapy, and outcome of immune-mediated thrombotic thrombocytopenic purpura at population level in Germany
Source: Res Pract Thromb Haemost. 2026 Mar 4;10(2):103406. doi: 10.1016/j.rpth.2026.103406 (PMC13049508; doi:10.1016/j.rpth.2026.103406)
Supplement: Supplementary Material [file mmc1.pdf]

Supplementary Appendix

to

Epidemiology, therapy approaches and acute outcome of immune-mediated thrombotic- thrombocytopenic purpura (iTTP) in Germany - a retrospective observational study at population level

Table of contents

Section

Table S1. ICD-10-GM codes used in the analyses. 3

Table S2. OPS codes used in the analyses. 4

Table S3. Hospital institution codes used to identify iTTP patients in university hospitals. 4

Figure S1. Geographical distribution of iTTP episodes treated in German hospitals between 2011 and 2021. 5

Figure S2. Age distribution of acute iTTP patients in German hospitals between 2011 and 2021. 6

Figure S3. Comorbidities of acute iTTP patients in German hospitals between 2011 and 2021. 7

Figure S4. Seasonal distribution of acute iTTP patients in German hospitals between 2011 and 2021. 8

Supplementary Table 1. ICD-10-GM codes used in the analyses.

**comorbidities**	**corresponding ICD-10-GM codes**
cerebrovascular disease	I60 - I69
chronic obstructive pulmonary disease (COPD)	J44
dementia	F00 - F03; G30
depression	F32 - F33
diabetes mellitus	E10 - E14
cancer	C00 - C97 exclusive C44
congestive heart failure (CHF)	I50
coronary heart disease (CHD)	I20 – I25
peripheral arterial disease (PAD)	I70.2 exclusive. I70.26
**organ system dysfunctions**	
cardiovascular	I95.9<br>R57.9<br>R57.8<br>I95.9<br>R57.9<br>R57.8
central nervous system	F05<br>G93.1<br>G93.4<br>R40<br>F05<br>G93.1<br>G93.4<br>R40
hematologic	D65<br>D68.8<br>D68.9<br>D69.5<br>D69.6<br>D65<br>D68.8<br>D68.9<br>D69.5<br>D69.6
hepatic	K72.0<br>K76.2<br>K72.7-!<br>K76.3<br>K72.0<br>K76.2<br>K72.7-!<br>K76.3
metabolic	E87.2<br>E87.2
renal	N17<br>N19<br>N17<br>N19
respiratory	J96<br>J96.9<br>J80<br>J98.4<br>R06.0<br>R06.8<br>J96.0<br>J96.9

	J80<br>J98.4<br>R06.0<br>R06.8
multi-organ dysfunction	R65.1!<br>R57.2<br>R65.1!<br>R57.2<br>or any combination of more than one of the above organ systems
**vascular complications**	
myocardial infarction	I21-24
stroke	I64<br>I63
other vascular occlusion	I74<br>H34<br>K55.0<br>N28.0
**Exclusion criteria / possible other causes of TMA**	
hemolytic uremic syndrome	D59.3
stem cell transplant	Z94.80, Z94.81
systemic lupus erythematosus (SLE)	M32
anti phospholipid syndrome	D68.6
hemolysis, elevated liver enzymes, and low platelet (HELLP) syndrome	O14.2

ICD-10-GM = International Classification of Diseases 10th Revision German Modification; TMA = thrombotic microangiopathy

Supplementary Table 2. OPS codes used in the analyses.

procedure	corresponding OPS codes
caplacizumab	6-00b.5
plasma exchange (performed procedures 1 – 50+)	8-820.00 - 8-820.09, 8-820.0a - 8-820.0w
intensive care treatment	8-980, 8-98d, 8-98f
mechanical ventilation	8-70 – 8-72
renal replacement therapy	8-853 – 8-857
rituximab	6-001.h, 6-001.j

OPS = "*Operationen- und Prozedurenschlüssel*", German adaption of the international classification of procedures in medicine

Supplementary Table 3. Hospital institution codes used to identify iTTP patients in university hospitals.

260102343	260820466	260510906	260950567	260610279
260102343	260820569	260530012	260960079	260612124
260200013	260832299	260530103	260970015	260620431
260310378	260840108	260530283	261000386	260730161
260320597	260840200	260550131	261101015	261401052
260340740	260913195	260590037	261300152	261500289
260510018	260914050	260590457	261300425	261500702
260510381	260930608	260591608	261401030	261600736

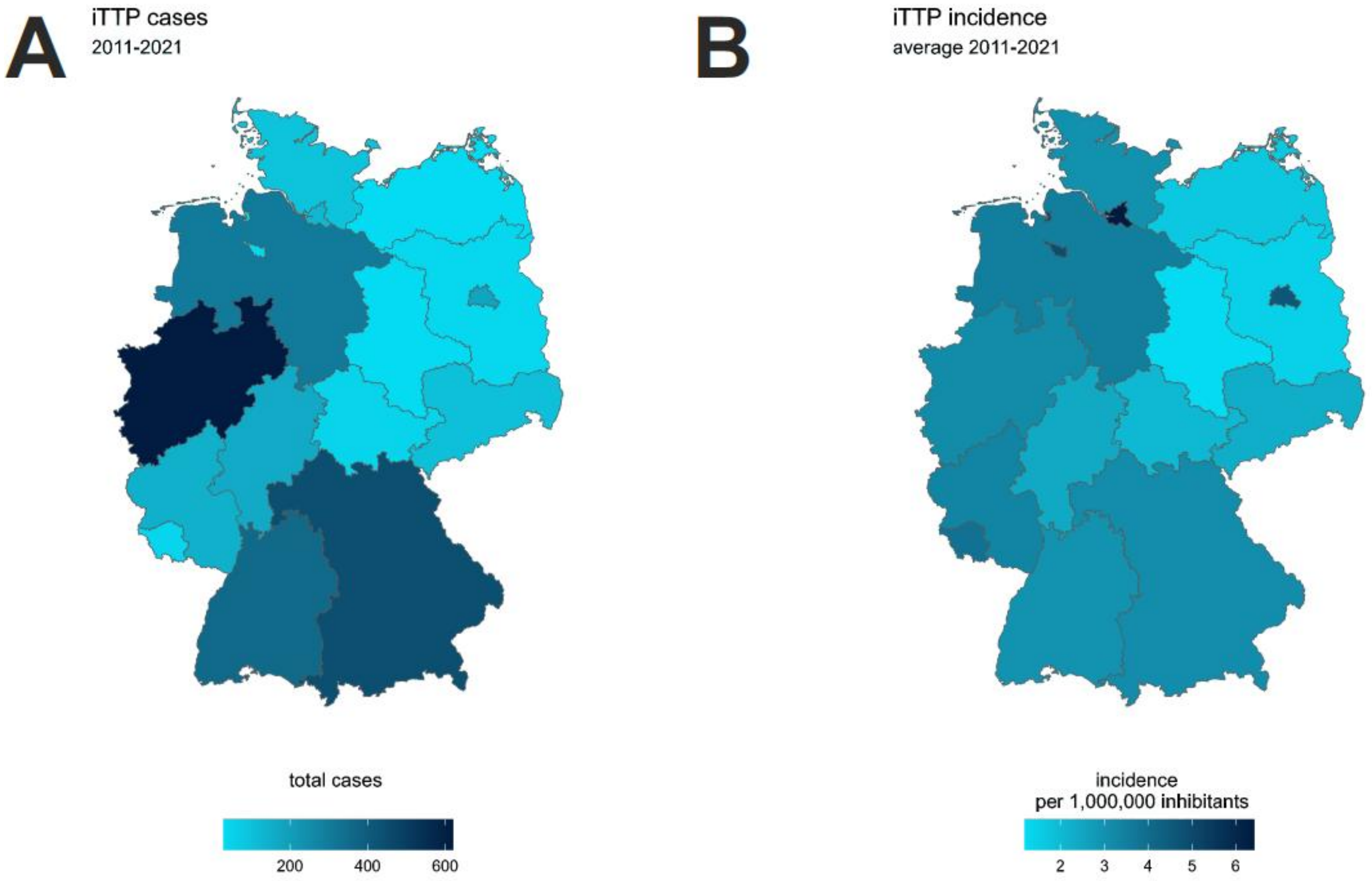

Supplementary Figure 1. Geographical distribution of iTTP episodes treated in German hospitals between 2011 and 2021.

The figure shows the distribution of iTTP episodes treated in the observation period across the various German federal states, with **A)** the distribution of cases by absolute numbers and **B)** the average annual incidence per 1,000,000 inhabitants in the federal states during the observation period.

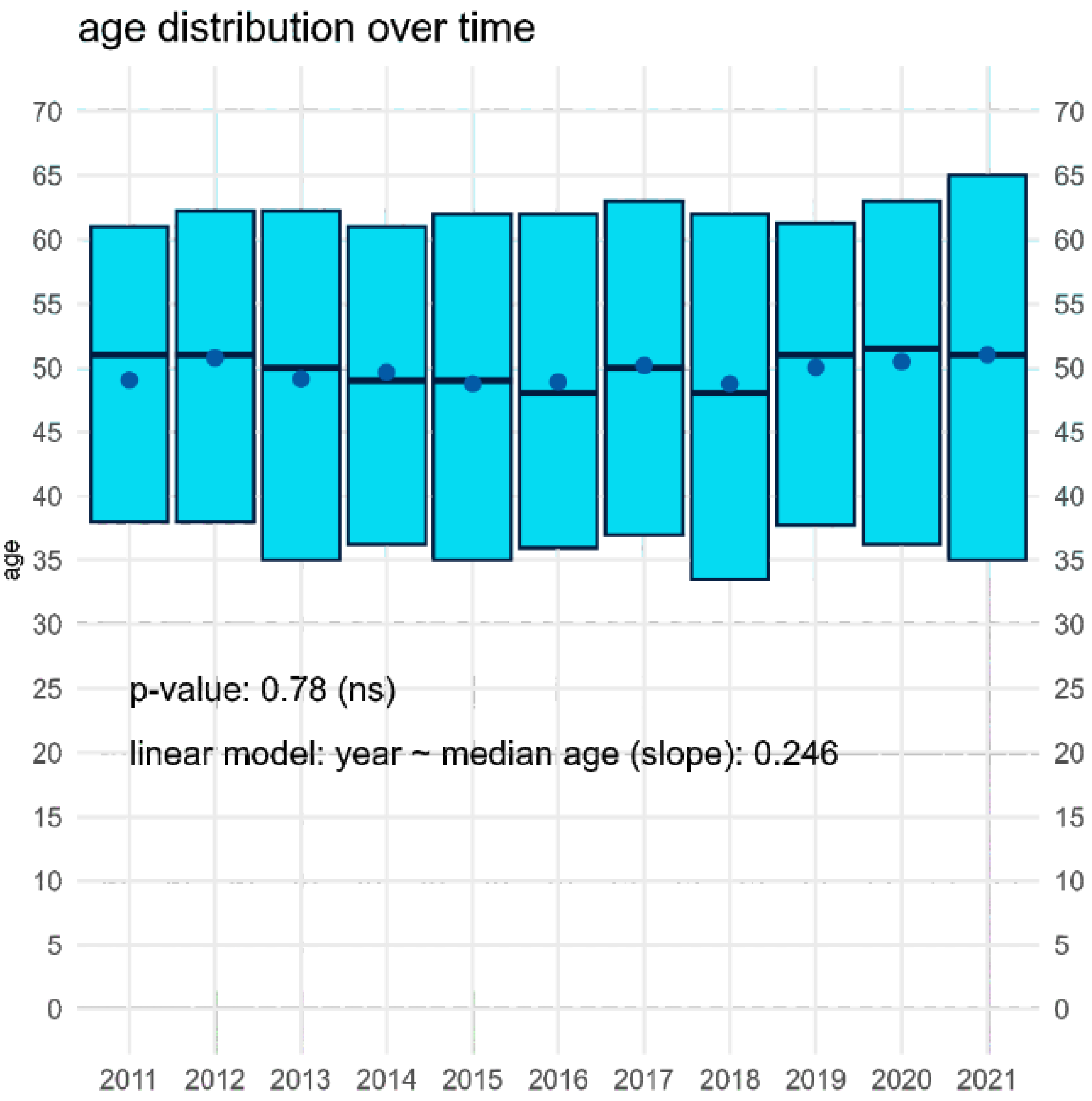

Supplementary Figure 2. Age distribution of acute iTTP patients in German hospitals between 2011 and 2021.

The figure shows the annual age distribution of acute iTTP patients in German hospitals with mean (point), median (horizontal bar) and interquartile range (box limits). Linear regression analysis did not show a significant trend in age distribution during the observation period.

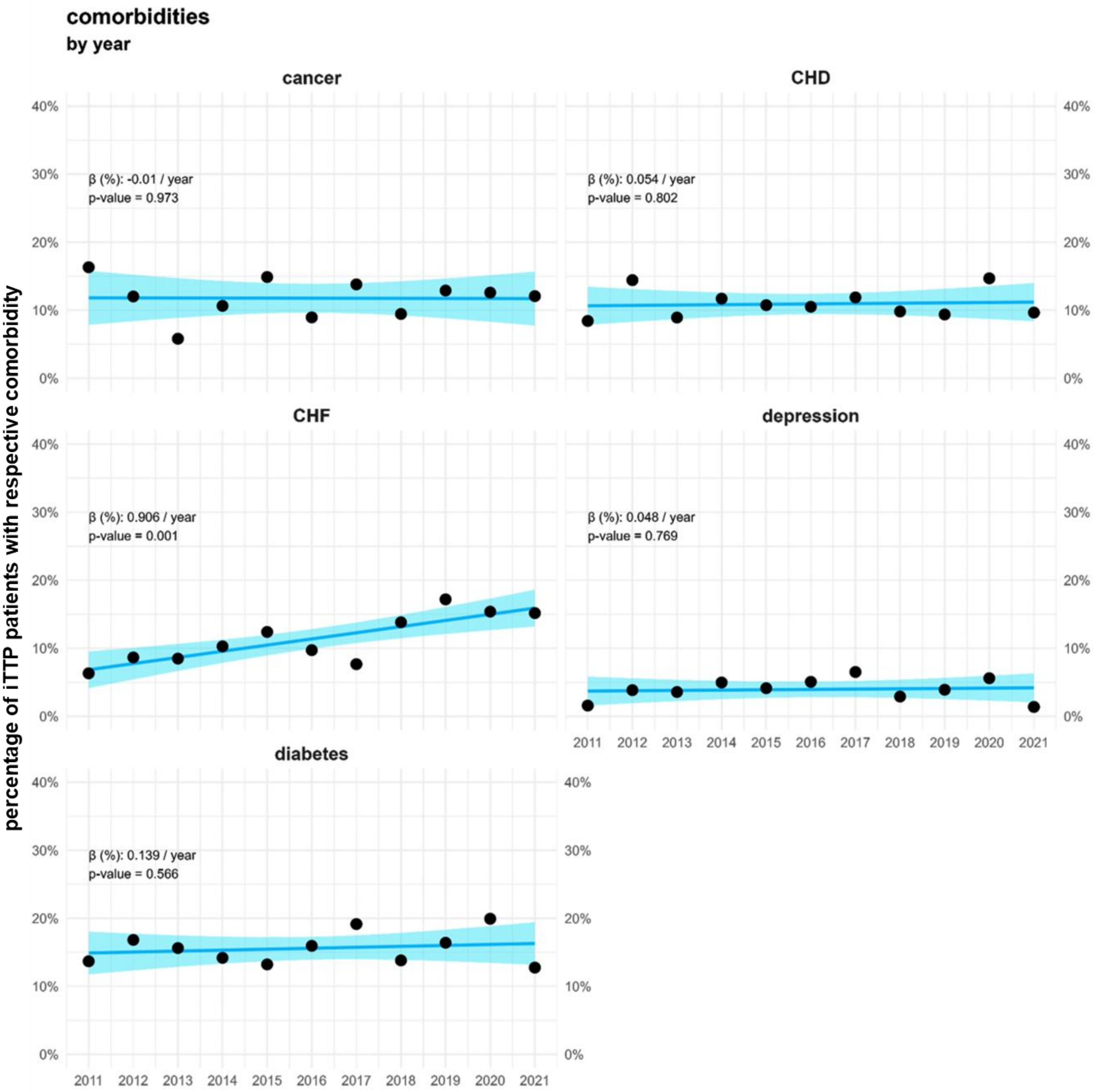

Supplementary Figure 3. Comorbidities of acute iTTP patients in German hospitals between 2011 and 2021.

The figure shows the known comorbidities of acute iTTP patients in German hospitals, namely cancer, coronary heart disease (CHD), congestive heart failure (CHF), depression and diabetes. Linear regression analyses showed a significant trend only for CHF, which increased during the observation period.

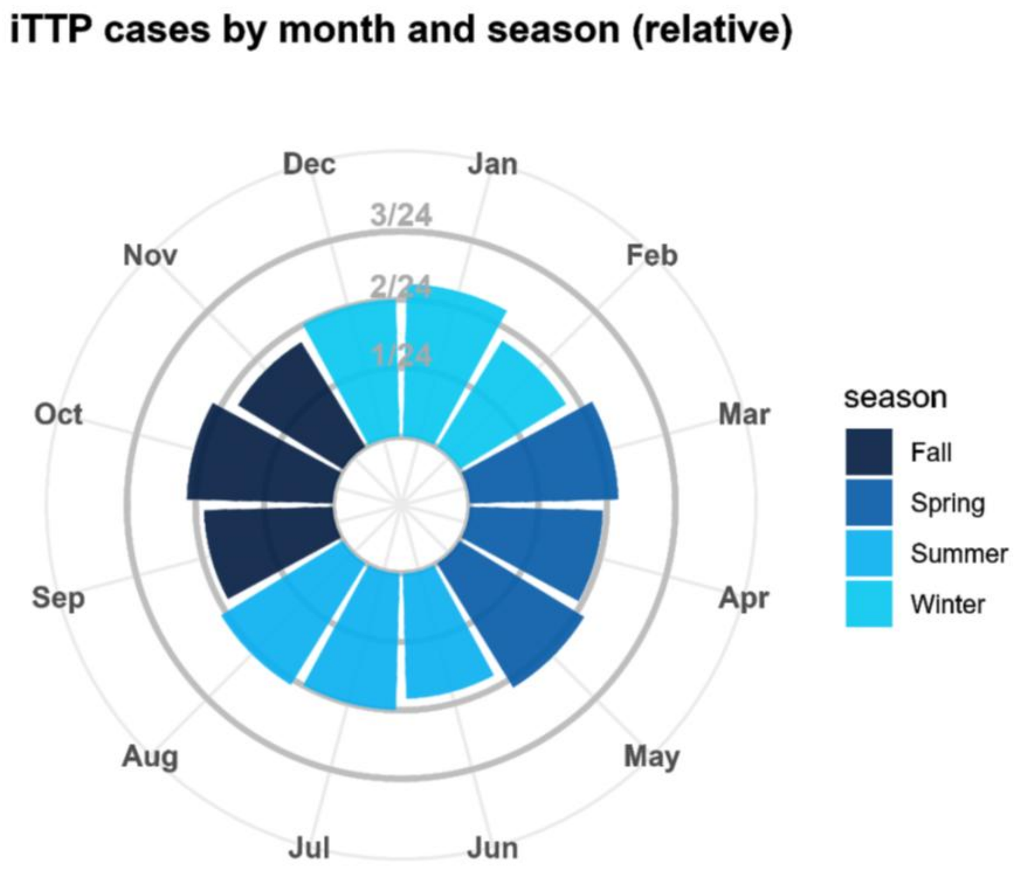

Supplementary Figure 4. Seasonal distribution of acute iTTP patients in German hospitals between 2011 and 2021.

The figure shows the relative distribution of acute iTTP episodes in German hospitals during the observation period, broken down into the different months of the year in circular bar plots grouped by season. The scale is in 1/24 steps as a monthly occurrence of 2/24 (=1/12) of iTTP episodes would represent an even distribution troughout the year. The reference date was the day of hospital admission.
